# Effect of dietary protein intake on the body composition and metabolic parameters of neutered dogs

**DOI:** 10.1017/jns.2017.41

**Published:** 2017-08-18

**Authors:** Iris Mayumi Kawauchi, Juliana Toloi Jeremias, Paula Takeara, Danilo Ferreira de Souza, Júlio Cesar de Carvalho Balieiro, Karina Pfrimer, Marcio Antonio Brunetto, Cristiana Fonseca Ferreira Pontieri

**Affiliations:** 1 Grandfood Indústria e Comércio Ltda (PremieR Pet), Dourado, São Paulo, Brazil; 2 School of Veterinary Medicine and Animal Science (FMVZ), University of Sao Paulo (USP), Av. Duque de Caxias Norte, 225, Pirassununga, 13635-900, SP, Brazil; 3 Ribeirão Preto Medical School (FMRP), University of São Paulo (USP), Ribeirão Preto, São Paulo, Brazil

**Keywords:** Canine food, Castration, Energy requirements, Diet protein concentration, Neutering, IGF-1, insulin-like growth factor-1, ME, metabolisable energy, MER, maintenance energy requirement, mld, minimum limit of detection, P60, diet containing 59·7 g protein/1000 kcal (4184 kJ), P94, diet containing 94·0 g protein/1000 kcal (4184 kJ)

## Abstract

Neutering is a common veterinary recommendation and is often associated with obesity development. Thus, the aim of the present study was to evaluate the effects of two different amounts of protein intake by neutered dogs regarding maintenance energy requirement (MER), body composition, and biochemical and hormonal parameters. A total of fourteen healthy adult dogs were fed either a diet containing 59·7 g protein/1000 kcal (4184 kJ) (P60) or a diet with 94·0 g protein/1000 kcal (4184 kJ) (P94) for 26 weeks after neutering to maintain their body weight prior to neutering. A mixed model was fitted to verify diet, time and diet × time interaction effects on biochemical parameters, serum concentrations of insulin, glucagon, leptin and insulin-like growth factor-1 (IGF-1). MER and the body composition data were evaluated within diets (paired *t* test) and within times (unpaired *t* test). A time effect was found for fructosamine, TAG, total lipids and IGF-1 serum concentrations. The diet × time interaction was significant for glucagon (*P* < 0·05). No differences between diets in the MER within each time were found. However, there was a reduction in the MER of dogs fed the P60 diet 26 weeks after neutering (*P* = 0·042). The fat body mass of dogs fed the P60 diet increased (*P* < 0·05) after neutering, even without a body-weight change. Some of the biochemical parameters changed over time, but all remained within the normal range. For the period evaluated in the present study, a diet with 94·0 g of protein/1000 kcal (4184 kJ) metabolisable energy seems to be a beneficial nutritional strategy to maintain the MER and the body composition of dogs after neutering.

It is estimated that over one-third of dogs in developed countries are overweight or obese, and, depending on the study, the proportion ranges from 33 to 58 %^(^^1^^–^^5^^)^. As in humans, pet obesity may be attributed to many factors, including genetics, sedentary lifestyle, neutering and overconsumption of energy-dense foods^(^^6^^,^^7^^)^. Neutering is a common veterinary recommendation in developed countries, and it is possible that some modifications after neutering occur, such as a decrease in basal metabolism^(^^8^^)^, impairment in the control of food intake^(^^9^^,^^10^^)^ as observed in cats, and/or a decrease in physical activity as suggested in dogs^(^^11^^,^^12^^)^. The influence of dietary composition has been previously studied in cats^(^^13^^)^, as well as in dogs^(^^14^^)^. Schauf *et al.*^(^^14^^)^ studied the effects of neutering and dietary fat and carbohydrate content on the hormones related to satiety in dogs. The authors observed no effect of neutering on food intake or the concentrations of ghrelin, insulin, cholecystokinin and total peptide YY. Moreover, a high-carbohydrate diet tended to increase the levels of the anorexigenic hormones cholecystokinin and total peptide YY.

One study reported that feeding a high-protein diet (117 g of protein/1000 kcal (4184 kJ) metabolisable energy (ME)) decreased the energy restriction needed for weight loss in obese cats and resulted in an increase in their energy requirements during weight maintenance^(^^15^^)^. However, no similar information was found in the literature for dogs regarding weight loss or after neutering.

Thus, the aim of the present study was to evaluate the effects of two different amounts of protein intake by neutered dogs considering maintenance energy requirement (MER), body composition, and biochemical and hormonal parameters.

## Materials and methods

The study was conducted at the PremieR Pet Nutritional Development Centre, Dourado, São Paulo, Brazil, and all experimental procedures were previously approved by its ethics and animal welfare committee.

### Animals, diets and experimental design

A total of fourteen healthy young adult dogs, nine males and five females, were divided into two groups balanced for breed (four beagles, two Yorkshire terriers and one golden retriever), age (1·9 (sd 0·156) years old), body weight (11·56 (sd 2·283) kg) and body condition score (4·1 (sd 0·3) on a nine-point scale^(^^16^^)^).

A commercial extruded (dry-type) diet recommended for adult dogs with 94·0 g of protein/1000 kcal (4184 kJ) ME (P94) was provided to one of the groups. The other group of dogs consumed a similar diet with the same ingredients but with a lower protein content, 59·7 g of protein/1000 kcal (4184 kJ) ME (P60). The diets were formulated to maintain the proportion between protein sources, and presented a similar concentration of nutrients, except for the protein and N-free extract. Both diets met the nutritional requirements recommended by the literature^(^^17^^)^ for adult dogs at maintenance (Supplementary Table S1).

During the study, the dogs had 2 h of voluntary physical activity in groups in a grassy area (30 × 50 m^2^) and remained in kennels (2·0 × 5·6 m^2^) for the rest of the day in pairs. The dogs had free access to water and were fed twice per d (07.00 and 15.00 hours). Bowls were removed after 30 min, and any food remaining was weighed and recorded.

The ME of each diet was determined in a digestibility trial through the total faecal collection method in accordance with the Association of American Feed Control Officials^(^^18^^)^ guidelines. Another group of eight healthy adult dogs was used for each evaluation.

The study was divided into two phases: (1) 4 weeks before neutering, to determine the initial body weight and initial MER; and (2) 26 weeks after neutering. During the study, dogs were weighed weekly, and the amount of diet offered was reassessed and adjusted, when needed, to maintain the body weight that was previously determined before neutering. The MER was then determined before neutering and 26 weeks after neutering.

Blood samples from dogs were collected for biochemical, hormonal and body composition evaluations. Dogs were fasted overnight, and blood samples were collected from the cephalic vein prior to feeding. Biochemical parameters were analysed after obtaining the serum samples. For hormonal and body composition evaluations, the serum was stored at −70°C until the analyses were performed.

Fructosamine, cholesterol, TAG and total lipids were measured before neutering and at 4 and 26 weeks after neutering. These parameters were measured by colorimetric methods using specific commercial kits (Labtest Diagnóstica S.A.). The assays were performed in accordance with the manufacturer's protocols in the PremieR Pet research laboratory (Dourado, SP, Brazil).

Circulating serum concentrations of insulin, leptin, glucagon and insulin-like growth factor-1 (IGF-1) were measured before neutering and at 4, 12 and 26 weeks after neutering. Insulin, leptin and glucagon were measured using a Luminex-based assay (Milliplex^®^ Canine Gut Hormone Magnetic Panel; Millipore). The intra- and inter-assay CV were 1 and 7 % for insulin (minimum limit of detection (mld) = 52·8 pg/ml), 1 and 8 % for leptin (mld = 80·6 pg/ml), and 2 and 13 % for glucagon (mld = 30·7 pg/ml). Serum IGF-1 was measured using an enzymic immunoassay (Enzyme-linked Immunosorbent Assay Kit; Uscn Life Science Inc.). The intra- and inter-assay CV were 10 and 12 % (mld = 3·5 ng/ml). Assays were performed in accordance with the manufacturer's protocols at the Institute Genese of Scientific Analyses (IgAc, São Paulo, Brazil).

Body composition was determined by the ^2^H oxide dilution method before neutering and at 26 weeks after neutering, according to the methodology described by Ferrier *et al*.^(^^19^^)^ and Brunetto *et al.*^(^^20^^)^.

### Statistical analysis

All data were found to comply with the assumptions of ANOVA models. An analysis for repeated measures using the mixed procedure (PROC MIXED), including in the model the effects of diet, time and their interaction (diet × time) as fixed effects and animals as the random effect, was performed. The data are presented as means and standard errors. The MER and the body composition data were evaluated within diets by a paired *t* test and within times (before and 26 weeks after neutering) by unpaired *t* test. Values of *P* < 0·05 were considered significant. Data were analysed using Statistical Analysis Systems software version 9.3 (SAS Institute).

## Results

No significant differences were observed in diet, time or diet × time interaction for fasting serum concentrations of cholesterol, insulin and leptin (*P* > 0·05). In the present study, no differences were found between diets regarding the glucose and insulin serum concentrations measured at fasting and at 15, 30, 45, 60, 120, 180, 240, 300 and 360 min after feeding (data not shown).

Modifications occurred over time in biochemical and hormonal parameters. A significant time effect was observed for IGF-1, fructosamine, TAG and total lipids (*P* < 0·05), but all remained within the normal range. The diet × time interaction was significant only for the glucagon serum concentrations (*P* < 0·05) (Table 1).

**Table 1.** Fasting serum concentrations of fructosamine, TAG, cholesterol, total lipids, insulin, leptin, glucagon and insulin-like growth factor-1 (IGF-1) of dogs fed diets containing different levels of protein before and after neutering (Mean values with their standard errors for *n* 7 dogs per diet)

			Weeks after neutering								
	Before neutering		4		12		26		*P* > F*		
Diet	Mean	se	Mean	se	Mean	se	Mean	se	Diet	Time	Diet × time
Serum fructosamine (μmol/l)											
P60	228·6	9·83	203·6	9·83			266·0	11·41	0·332	<0·001	0·059
P94	245·7	9·83	232·4	9·83			251·2	9·83			
Serum TAG (mg/dl)											
P60	50·3	8·08	59·0	8·08			73·2	8·44	0·175	<0·001	0·112
P94	39·4	8·08	47·4	8·08			48·7	8·08			
Serum cholesterol (mg/dl)											
P60	166·1	11·86	186·0	11·86			171·0	12·99	0·284	0·115	0·660
P94	157·1	11·86	164·6	11·86			151·9	11·86			
Serum total lipids (mg/dl)											
P60	441·0	13·07	466·1	13·07			464·5	14·12	0·119	0·038	0·418
P94	423·7	13·07	436·9	13·07			427·1	13·07			
Serum insulin (pg/ml)											
P60	338·7	65·29	252·7	65·29	302·6	65·29	384·4	76·90	0·313	0·562	0·917
P94	263·3	65·29	245·57	65·29	247·6	65·29	292·7	65·29			
Serum leptin (pg/ml)											
P60	491·4	263·90	467·6	263·90	427·8	186·60	346·0	312·25	0·613	0·789	0·687
P94	390·2	312·25	380·2	312·25	819·7	201·55	546·6	312·25			
Serum glucagon (pg/ml)											
P60	63·8^a,A,B^	10·94	72·6^a,A^	10·94	70·0^a,A^	10·94	53·7^a,B^	11·28	0·883	0·192	0·043
P94	66·5^a,A^	10·94	64·0^a,A^	10·94	69·4^a,A^	10·94	69·1^a,A^	10·94			
Serum IGF-1 (ng/ml)											
P60	37·7	6·18	27·7	6·18	21·3	6·18	18·7	6·79	0·726	0·008	0·461
P94	29·8	6·18	21·8	6·18	20·2	6·18	23·2	6·18			

P60, diet with 59·7 g of protein/1000 kcal (4184 kJ) metabolisable energy; P94, diet with 94·0 g of protein/1000 kcal (4184 kJ) metabolisable energy.

^a,b^ Mean values within a column with unlike superscript letters were significantly different (*P* < 0·05).

^A,B^ Mean values within a row with unlike superscript letters were significantly different (*P* < 0·05; Tukey's test).

* A *P* value < 0·05 indicates a significant difference.

No differences were found between diets in the MER within each time (before and 26 weeks after neutering). However, there was a reduction in the MER of dogs fed the P60 diet 26 weeks after neutering (*P* = 0·042).

As the experimental design of the study, the dogs were fed to maintain their body weight before neutering. Therefore there was no modification in this parameter for both diets (*P* > 0·05). There were no differences between diets for lean body mass (*P* > 0·05). Fat body mass between diets before neutering was the same. However, 26 weeks after neutering, the dogs fed the P60 diet presented higher fat body mass (kg) than dogs fed the P94 diet (*P* = 0·037) (Table 2).

**Table 2.** Maintenance energy requirement (MER) and body composition of dogs fed diets containing different levels of protein, before neutering and 26 weeks after neutering (Mean values with their standard errors for *n* 7 dogs per diet)

	Before neutering		26 weeks after neutering		
Diet	Mean	se	Mean	se	P*
MER (kcal/kg BW^0·75^)					
P60	65·0	3·43	52·4	3·54	0·042
P94	64·1	3·43	59·7	3·68	0·414
*P*	0·876		0·621		
MER (kJ/kg BW^0·75^)					
P60	272·0	14·4	219·2	14·8	0·042
P94	268·2	14·4	249·8	15·4	0·414
*P*	0·876		0·621		
Body weight† (kg)					
P60	12·7	3·50	12·9	3·50	0·991
P94	11·5	3·24	11·5	3·24	0·969
*P*	0·726		0·780		
Fat body mass (kg)					
P60	1·9	0·57	2·3	0·57	0·009
P94	1·6	0·52	1·8	0·52	0·760
*P*	0·400		0·037		
Lean body mass (kg)					
P60	10·9	3·68	10·7	3·68	0·970
P94	9·9	3·36	9·6	3·36	0·962
*P*	0·715		0·716		
Fat body mass (%)					
P60	14·7	1·52	18·1	1·52	0·026
P94	15·2	1·39	18·1	1·39	0·250
*P*	0·973		0·430		
Lean body mass (%)					
P60	85·3	1·52	81·6	1·52	0·026
P94	84·8	1·39	81·9	1·39	0·250
*P*	0·973		0·430		

P60, diet with 59·7 g of protein/1000 kcal (4184 kJ) metabolisable energy; P94, diet with 94·0 g of protein/1000 kcal (4184 kJ) metabolisable energy.

* A *P* value < 0·05 indicates a significant difference.

† As the experimental design of the study, the dogs were fed to maintain their body weight before neutering.

No difference was found in body composition between times (before and 26 weeks after neutering) of dogs fed the P94 diet (*P* > 0·05). However, the kg and the proportion of fat body mass in percentage of dogs fed the P60 diet increased (*P* = 0·009 and *P* = 0·026, respectively) after neutering (Table 2).

## Discussion

Differences were found 26 weeks after neutering. There were no differences between diets for MER. However, when the data were analysed over time for each diet, a reduction at the end of the study of 19·4 % in MER was necessary so that the dogs fed the P60 diet could maintain their body weight. Jeusette *et al.*^(^^21^^)^ observed a 30 % MER reduction 6 months after neutering in beagles fed a diet that had a similar protein content to the P60 diet (58·8 *v.* 59·7 g of protein/1000 kcal (4184 kJ), respectively). This large reduction verified by Jeusette *et al.*^(^^21^^)^ was probably due to the differences in energy requirements between sexes, since these authors determined this value in a group containing only females. The reduction in the MER after neutering may be an indicator of an important interaction among reproductive status, nutrition and MER and should be better studied.

At 26 weeks after neutering, the fat body mass of dogs fed the P60 diet was greater than the fat body mass of dogs fed the P94 diet (2·3 *v.* 1·8 kg, respectively). Moreover, when the data were evaluated over time for the P60 diet, an increase in the fat body mass from 1·9 kg before neutering to 2·3 kg was found 26 weeks after neutering, which corresponds to a 21 % increase even without body-weight modification. The increase in the fat body mass percentage indicates an important modification in the body composition of dogs fed the P60 diet.

In the present study, the activity level of dogs was not measured; therefore, it was not possible to determine behaviour differences before and after neutering or between the diets. Schauf *et al.*^(^^14^^)^ observed an increased risk of body-weight gain in dogs after neutering and attributed this effect to a decrease in energy expended in physical activity and not to an increased level of energy intake.

Studies of cats have demonstrated a reduction in MER after neutering. Mitsuhashi *et al.*^(^^22^^)^ observed a reduction of 25 % in the MER 12 weeks after neutering. The MER reduction after neutering is a relevant modification because it increases the difficulty of nutritional management of owned animals, mainly because this modification is silent and develops relatively quickly. Moreover, in clinical practice in Brazil, no common veterinary recommendations exist for the owners regarding food adjustments after neutering. Therefore, the utilisation of a higher-protein diet for dogs, such as the 94·0 g of protein/1000 kcal (4184 kJ) diet, after neutering may enable the owner to offer the same quantity that was offered before neutering with a lower risk of body fat accumulation.

The MER reduction after neutering results in a need for strict control of energy availability to maintain body weight, resulting in a reduction in food intake and, as a consequence, a reduction in the intake of protein and other nutrients. It is important to highlight that during the experimental period, dogs offered the P60 diet consumed the dietary protein recommended by the literature for adult dogs at maintenance^(^^17^^)^, but with the MER reduction, a more pronounced reduction in protein intake occurred than in dogs fed the P94 diet. It is possible that the protein intake by dogs fed the P60 diet was not sufficient to maintain their body composition. Laflamme & Hannah^(^^23^^)^ observed that the current literature recommendations^(^^17^^,^^18^^)^ may not provide adequate protein to support the lean body mass of adult cats. More studies are necessary to determine whether neutered dogs require more protein in order to maintain their body composition than the current recommendations in the literature for adult dogs at maintenance.

After neutering, modifications in biochemical parameters occurred within the normal range and indicated little or no clinical relevance in the short term. However, longevity studies are needed to verify whether such changes will have a significant effect throughout the life of neutered and healthy dogs, since the changes may correspond to primary modifications in the metabolism of carbohydrates and lipids.

The serum concentration of IGF-1 decreased significantly after neutering. Decreasing serum IGF-1 levels were previously observed in dogs with energy restriction^(^^24^^,^^25^^)^, in humans^(^^26^^)^ and in puppies^(^^27^^)^ with protein restriction and after neutering in dogs fed a diet that was similar in protein content to the P60 diet^(^^28^^)^. It is important to note that the dogs in the present study were not under strict energy restriction, since their body weight was maintained throughout the study. The dogs were also not under protein restriction, as both diets met the protein recommendations for adult dogs at maintenance^(^^17^^)^. Reductions in IGF-1 serum concentrations and IGF-1 signalling have been associated with health and life span increases in flies, mice and other animal models. However, it is still unknown whether this phenomenon occurs in dogs.

### Conclusions

For the period evaluated in this study, a diet with 94·0 g of protein/1000 kcal (4184 kJ) ME seems to be a beneficial nutritional strategy to maintain the MER and body composition of dogs after neutering. More studies are needed to verify the impact of the different levels of protein intake to prevent obesity after neutering.

## Supplementary material

For supplementary material accompanying this paper visit http://dx.doi.org/10.1017/jns.2017.41. click here to view supplementary material
