# Supplementary material for: Effect of dietary protein intake on the body composition and metabolic parameters of neutered dogs
Source: J Nutr Sci. 2017 Aug 18;6:e40. doi: 10.1017/jns.2017.41 (PMC5672309; doi:10.1017/jns.2017.41)
Supplement: Supplementary file 1 [file S2048679017000416sup001.doc]

**Supplementary Table S1.** Ingredient composition, analysed chemical composition, coefficients of total tract apparent digestibility of nutrients and energy content of diets containing different levels of protein

| **Item** | **Diet** | |
| --- | --- | --- |
|  | **P60^1^, g/kg** | **P94^2^, g/kg** |
| Ingredient composition, g/kg, as-fed basis | | |
| | Chicken by-product meal, pork isolated protein, soy isolated protein, brewer’s yeast, fish meal, egg | | --- | | 154 | 392 |
| Maize, rice | 450 | 258 |
| Barley, pea flour | 120 | 120 |
| Sugarcane fibre, beet pulp | 100 | 100 |
| Pork fat, chicken fat, fish oil | 84 | 58 |
| Dicalcium phosphate | 20 |  |
| Minor ingredients^2^ | 72 | 72 |
| Total | 1,000 | 1,000 |
| Analysed chemical composition, g/1,000 kcal of ME | | |
| Crude protein | 59.7 | 94.0 |
| Nitrogen-free extract | 136.1 | 107.1 |
| Crude fibre | 11.8 | 8.8 |
| Acid-hydrolysed fat | 36.9 | 38.0 |
| Coefficient of total tract apparent digestibility^3^, % | | |
| DM | 81.0 | 78.3 |
| Organic matter | 83.6 | 80.8 |
| Crude protein | 84.5 | 85.6 |
| Nitrogen-free extracts | 86.0 | 81.7 |
| Fat | 96.1 | 94.3 |
| Energy | 84.5 | 82.1 |
| Energy content, kcal/g diet | | |
| Gross energy^3^ | 4.6 | 4.7 |
| Metabolizable energy^4^ | 3.6 | 3.5 |

^1^Extruded (dry-type) diet for adult dogs. Diet with 59.7 g of protein/1,000 kcal ME.

^2^Commercial extruded (dry-type) diet indicated for adult dogs (Ambientes Internos Cães Castrados, PremieR pet, Dourado, São Paulo, Brazil). Diet with 94.0 g of protein/1,000 kcal ME.

^2^Potassium chloride, sodium chloride, methionine, lysine, carnitine, mannan-oligosaccharides, sodium hexametaphosphate, vitamin and mineral supplements, palatability enhancer, yucca extract, adsorbent of mycotoxins, mould inhibitor and antioxidant.

^3^Determined by bomb calorimetry.

^4^Determined in digestibility trials with dogs *(n =* 8 dogs per diet) by the total faecal collection method in accordance with the American Association of Feed Control Officials^(18)^ protocol.
